# New Trends in Precision Medicine: A Pilot Study of Pure Light Scattering Analysis as a Useful Tool for Non-Small Cell Lung Cancer (NSCLC) Diagnosis

**DOI:** 10.3390/jpm11101023

**Published:** 2021-10-13

**Authors:** Domenico Rossi, David Dannhauser, Bianca Maria Nastri, Andrea Ballini, Alfonso Fiorelli, Mario Santini, Paolo Antonio Netti, Salvatore Scacco, Maria Michela Marino, Filippo Causa, Mariarosaria Boccellino, Marina Di Domenico

**Affiliations:** 1 Center for Advanced Biomaterials for Healthcare@CRIB, Istituto Italiano di Tecnologia (IIT), Largo Barsanti e Matteucci 53, 80125 Naples, Italy; domenico.rossi@iit.it (D.R.); paoloantonio.netti@unina.it (P.A.N.); 2 Interdisciplinary Research Centre on Biomaterials (CRIB), Dipartimento di Ingegneria Chimica, dei Materiali e della Produzione Industriale, Università degli Studi di Napoli “Federico II”, Piazzale Tecchio 80, 80125 Naples, Italy; david.dannhauser@unina.it (D.D.); causa@unina.it (F.C.); 3 Department of Experimental Medicine, University of Campania “Luigi Vanvitelli”, 80138 Naples, Italy; bmnastri@gmail.com; 4 School of Medicine, University of Bari “Aldo Moro”, 70124 Bari, Italy; 5 Department of Precision Medicine, University of Campania “Luigi Vanvitelli”, 80138 Naples, Italy; mariamichela.marino@unicampania.it (M.M.M.); mariarosaria.boccellino@unicampania.it (M.B.); marina.didomenico@unicampania.it (M.D.D.); 6 Department of Translational Medical and Surgical Science, Thoracic Surgery Unit, University of Campania “Luigi Vanvitelli”, 80131 Naples, Italy; alfonso.fiorelli@unicampania.it (A.F.); mario.santini@unicampania.it (M.S.); 7 Department of Basic Medical Sciences, Neurosciences and Sense Organs, University of Bari “Aldo Moro”, 70124 Bari, Italy; 8 Department of Biology, College of Science and Technology, Temple University, Philadelphia, PA 19121, USA

**Keywords:** non-small cell lung cancer (NSCLC), circulating tumour cells (CTC), biophysical profile, clinical biochemistry, personalized medicine, medical biotechnologies, translational research, pure light scattering, machine learning

## Abstract

**Simple Summary:**

A reliable method for a fast diagnosis of non-small cell lung cancer (NSCLC) would greatly help in improving therapeutic success in personalized medicine approaches. Thus, in the present study, a new idea was proposed: a morphological single-cell analysis approach combined with a microfluidic device for liquid biopsy. The investigation of the NSCLC sample at different culturing times created the possibility of understanding the evolution of different cell types and their morphological changes, making the Circulating Tumour Cells (CTC) predominance against all other cell classes visible.

**Abstract:**

**Background:** To date, in personalized medicine approaches, single-cell analyses such as circulating tumour cells (CTC) are able to reveal small structural cell modifications, and therefore can retrieve several biophysical cell properties, such as the cell dimension, the dimensional relationship between the nucleus and the cytoplasm and the optical density of cellular sub-compartments. On this basis, we present in this study a new morphological measurement approach for the detection of vital CTC from pleural washing in individual non-small cell lung cancer (NSCLC) patients. **Materials and methods:** After a diagnosis of pulmonary malignancy, pleural washing was collected from nine NSCLC patients. The collected samples were processed with a density gradient separation process. Light scattering analysis was performed on a single cell. The results of this analysis were used to obtain the cell’s biophysical pattern and, later on, as basis for Machine Learning (ML) on unknown samples. **Results:** Morphological single-cell analysis followed by ML show a predictive picture for an NSCLC patient, screening that it is possible to distinguish CTC from other cells. Moreover, we find that the proposed measurement approach was fast, reliable, label-free, identifying and count CTC in a biological fluid. **Conclusions:** Our findings demonstrate that CTC Biophysical Profile by Pure Light Scattering in NSCLC could be used as a promising diagnostic candidate in NSCLC patients.

## 1. Introduction

Nowadays, cancer diagnosis and treatment mostly use solid biopsy from primary or secondary tumours. This procedure has several disadvantages both for the patient—who is the object of a real invasive surgery technique—and for the hospital, since this procedure is very expensive and time consuming. Such difficulties are particularly of interest when dealing with tumours with difficult surgical access and a high mortality rate, such as non-small cell lung cancer (NSCLC) [1,2], which is the main cause of death in men and the second one in women worldwide, with a 5-year overall survival rate of 20–25% for all stages [3]. This is due to the fact that, since occult micro metastases not even detectable with high-resolution imaging procedures can be present, patients in the early stages undergo surgery, but only 12% survive longer than 5 years. However, surgery is usually not indicated in advanced-stage patients for whom palliative treatment, including chemo and radiation therapy, is indicated [4]. The prognoses of NSCLC patients are highly different: stage IB–IIIA patients have a 20% probability of survival, while stage IIIB and IV patients are not operable. NSCLC patients can be characterized by two different histological types, which are adenocarcinoma (ADC) and squamous cell carcinoma (SCC), and the ADC is the most common. ADC presents itself with distant metastasis through inflammation, differently from SCC, which is associated with local tissue infiltration [3].

Only 5% of patients with lung cancer diagnosed at an advanced stage survive, while the survival rate significantly increases to 56% for those diagnosed at an early stage [5]. Since the mentioned difficulties, diagnosis techniques different from the current ones could improve the treatment success. Being able to have non-invasive, label-free and economic diagnostic tools represents the challenge of the last decade in the field of tumours, and the most interesting and promising are surely the liquid biopsy techniques. From this point of view, the searching of Circulating Tumour Cells (CTC) in biological fluids can represent the ideal tool for both early diagnosis and follow up analysis. CTC, in fact, are easily isolated from the blood or pleural effusion [4], with no risk and high patient compliance. Moreover, the clinical utility of CTC is not confined to their enumeration; in fact, CTC lend themselves well to molecular characterization such as proteomic, genomic or even mRNA and methylation analysis. Keeping in mind the considerable heterogeneity of NSCLC tumours, inclusion of a diverse range of targets for identification of CTC in NSCLC increases the probability of detection, thus providing insight into tumour burden and disease progression [6].

The first CTC studies were performed on patients with breast cancer and the first detection test was made through the EPithelial Immuno SPOT (EPISPOT) assay, which identifies them using an immunocytochemical labelling with cytokeratin-19. These studies have shown the importance of CTC from both a prognostic and therapeutic point of view, affirming the existence of an inverse correlation between their presence and progression-free survival [7,8,9,10,11]. CellSearch™ is nowadays the only method validated by the FDA for the detection of CTC in blood in patients with NSCLC. This method is based on the capture of the CTC with magnetic beads recognizing and binding the epithelial cell adhesion molecule (anti-EpCam) over them. However, CellSearch™ fails in detecting CTC that have undergone an epithelial–mesenchymal transition, which is a subpopulation of particular clinical interest in patients with NSCLC. Several other methods for the detection of CTC exist (ISET, Screen cell, etc.), but they are expensive, time consuming, complex in implementation and generally provide complicated and not-immediate data that require highly specialized personnel to be correctly interpreted [2].

**Figure 1:**
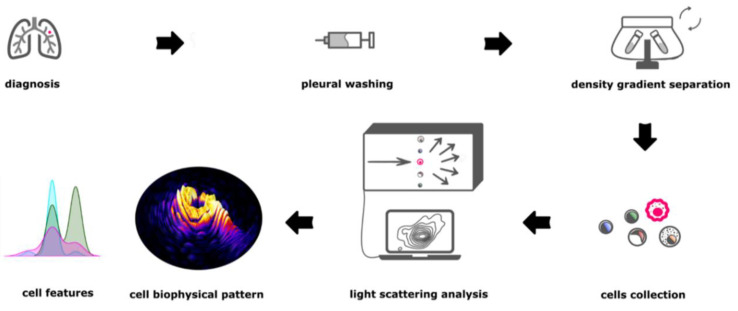
Schematic representation of the experimental approach. After a diagnosis of pulmonary malignancy, surgeon performs a pleural washing. The collected sample is processed with a density gradient separation process. Light scattering analysis is performed on single cell. Results of such analysis are used to find cells biophysical pattern and, later on, as basis for Machine Learning on unknown samples.

On this basis, we present in this study a new morphological measurement approach for the detection of vital CTC from pleural washing in patients with NSCLC (Graphical Abstract—Figure 1).

To accomplish this target, we performed label-free light scattering analysis of pleural washing and analysed our outcome with the help of machine learning, which enabled us to precisely characterize different cell types involved in an NSCLC event. Therefore, our results can significantly help in the diagnosis and therapeutic decisions of NSCLC events [2,4,12,13,14]. However, our presented morphological single cell analysis is able to reveal small structural modification of cells and therefore can retrieve several biophysical cell properties, such as the cell dimension, the dimensional relationship between the nucleus and the cytoplasm and the optical density of cellular sub-compartments [15,16,17,18]. In fact, our label-free measurement approach allows us to distinguish single-cell classes from other cells, such as red blood cells (RBC), lymphocytes, monocytes or macrophages.

A correct interpretation of CTC amount, finally, guarantees the administration of personalized therapies for each specific tumour stage (which directly correlates with the amount of CTC). The aim of this pilot study was to identify the biophysical patterns of CTC, from pleural washing of NSCLC patients, by means of the Pure Light Scattering analysis as an accurate pre-screening method so as to be able to trace a more detailed, rapid and clear diagnostic picture of tumour dynamics by evaluating whether in patients with NSCLC in the early stages, CTC are already present.

## 2. Materials and Methods

### 2.1. Pleural Washing Sample Collection

Pleural washing samples were collected from 9 NSCLC patients, previously already identified from pathologist examination. The study was conducted according to the guidelines of the Declaration of Helsinki and approved by the ethical committee of AOU Università degli Studi della Campania Luigi Vanvitelli, Protocol number 417 on 22 July 2014 entitled “*Il ruolo prognostico dei miRNA e delle cellule tumorali circolanti nei pazienti operati di neoplasia del polmone*”. This study was carried out according to the Helsinki declaration and informed written consent was obtained from all patients included in the study. In the present manuscript, data regarding medical history and clinical-instrumental work-up were assessed for each patient. Pleural washing collection and storage have been performed during surgical intervention, before any manipulation of the lung and cancer resection, also according, in some aspects of the technique, to previous studies [7]. 

### 2.2. Cell Collection

To collect potential CTC, we performed a density gradient separation on each sample. Since, we had an average of 40 to 60 mL of sample, within 3 h from the collection, each sample was layered on separation medium with density 1.077 (Ficoll) of an equal volume (ratio 1:1). Samples were centrifuged at 300 for 45 min, no brake. After the rotor of the centrifuged stopped, we collected the cells at the interface between sample fluid (at the bottom) and the Ficoll (at the top) with a Pasteur plastic sterile pipette. Depending on the state of the collected volume of cells, we evaluated to perform a washing with RBC lysis buffer (155 mM NH_4_Cl, 10 mM KHCO_3_, 0.1 mM EDTA) to eliminate possible RBC contaminants. Whenever the collected pellet of cells was visibly red, we added 10 mL of lysis for 5 min. Afterwards, samples were centrifuged at 200 for 10 min and re-suspended in a complete medium for circulant cells with the following composition: RPMI 1640, 10% FBS, 1% pen-strep, 1% L-Glutamine. For the whole time span needed for light scattering analysis—never longer than 2 h—cells were stored in the same complete medium and placed in a controlled ambience (37 °C, 5% CO_2_). Cells collected from NSCLC patients were cultured over time to select CTC in order to obtain a stable clone, which we labelled “training sample”. Such cultured cells provide the basis for subsequent CTC identification in other cell samples. Cells were cultured under controlled conditions (37 °C, 5% CO_2_) for seven weeks. This period of cell culture was necessary to obtain, in this pilot study, a stable clone from the isolated circulating cell, which allowed us to isolate the biophysical pattern of the labeled “training sample”. At three specific time steps, an aliquot of cells was collected to perform morphological single-cell analysis: time 0 (collection of NSCLC patient cells), time 1 (2 weeks of culturing), and time 2 (7 weeks of culturing). At each time step, cells were collected, centrifuged at 220 for 10 min and resuspended in complete RPMI medium.

### 2.3. Bright Field Microscope Evaluation

Before each cell measurement, we evaluated the state of cells and checked if the cell isolation procedure altered their membrane integrity, using a standard bright-field microscope (X81, OLYMPUS) with a 100X oil immersion objective. Moreover, quiescent cell dimension values were recorded and compared with in-flow obtained morphological single-cell results.

### 2.4. Microfluidic Device

Morphological single-cell measurements were performed with a microfluidic device, which first aligns cells in a single centreline and then enables the optical investigation of separated individual cells in flow [19,20,21,22,23]. In more detail, our microfluidic device is composed of two glass channels placed in a 3D-printed supporting structure. In general, cells are aligned in the centreline while passing the first glass channel, which is round shaped (Molex, inner radius R = 25 µm). Then, cells pass into a second observation channel, which is square shaped with a wider cross section of 400 × 400 µm (Vitrocom), where the previously obtained 3D alignment was preserved. Here, morphological single-cell analysis is performed. In fact, cell flow aligned at the centreline of a channel of the microfluidic chip thanks to a highly biocompatible viscoelastic alignment medium in which they were suspended. Such medium is composed of polyethylene oxide (PEO) powder diluted in phosphate-buffered saline (PBS) at 0.4 wt%.

The alignment probability to the centreline at the end of the first channel can be expressed by a dimensionless parameter $\theta =3Wi\beta^{2}\frac{L}{2R}>-ln\left(3.5\beta \right)$, with $Wi=2\lambda \bar{U}⁄2R$, where λ is the relaxation time (0.197 ms) of the fluid, $\bar{U}$ is the average fluid velocity (1496 µm s^−1^), $\beta =r_{1}⁄R$, a non-dimensional geometrical channel parameter, with $r_{1}$ the cell radius and L the channel length (0.35 m). All morphological single-cell measurements were performed with a flow rate of ~12 μm/s, in the observation channel obtaining a variable living cell detection throughput up to 1.2 cells/s.

### 2.5. Morphological Single Cell Analysis

**Figure 2:**
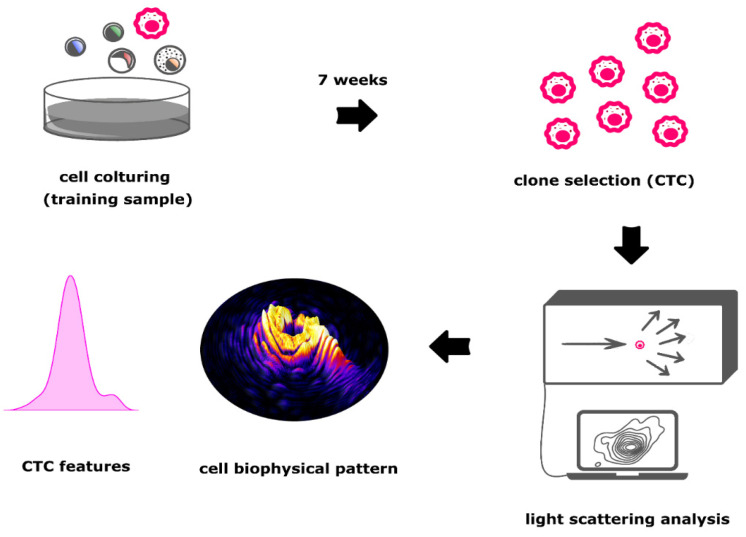
Flowchart of the generation of CTC clone and the consequent biophysical pattern to be used for the ML training.

We used a static light scattering approach, which collects diffraction patterns of scattered cells in a continuous angular range from 2° up to 30°, with a resolution of 0.1°. In detail, cells pass through a tightly collimated laser beam (λ = 632.8 nm), and then scattered light is collected and mapped onto a camera sensor (ORCA Flash 4.0, Hamamatsu Photonics K.K., Hamamatsu City, Shizuoka, Japan). The obtained scattering signal is processed to obtain a light-scattering profile (LSP). Such an LSP is analysed to retrieve four biophysical properties out of each single cell: Diameter (D), measured with a precision of 0.01 µm, Refractive Index (RI) of nucleus (RI_N_) and cytosol (RI_C_), both measured with a precision of 0.01, and nucleus over cytosol ratio (N/C-ratio), measured with a precision of 0.005. Collected experimental LSP are matched with a look-up table of more than 300,000 previously calculated theoretical scattering profiles, obtaining the values of each of the four biophysical properties to retrieve the searched for biophysical single cell properties (Figure 2).

### 2.6. Machine Learning

Machine Learning (ML) was implemented (MATLAB-R2020b, MathWorks Corp., Natick, MA, USA) in the measurement approach to identify red blood cells (RBC), T-lymphocytes (T), B-lymphocytes (B), monocytes (M), granulocytes (Macro) and CTC cells from experimental morphological single-cell data. In general, ML requires two steps: training of the test data and a test of the experimental data. The training step enables the ML to classify a cell class from another one. In more detail, an ML algorithm calculates the best matching set of cell properties for each cell type, by knowing the related cell type (labelled data). Afterwards, the previously trained ML algorithm is applied on unknown cells to predict its type and associated prediction accuracy. We tested the performance of different ML algorithms to find the fastest and most suitable one for our measurement approach. The quadratic support vector machine (SVM) was found to be the most suitable algorithm according to speed and accuracy, having an accuracy of 99.8%. As comparison, the fine Gaussian SVM-trained classifier showed an accuracy of 97.3%, and the linear SVM an accuracy of 99.5%, medium KNN 99.2% and kernel naive Bayes 92.2%. In more detail, we used our classifier with standardized data, a quadratic kernel function, box constraint level of 1 and multiclass method of one-vs.-one. Note that the ML algorithm was trained with data of isolated cell classes (labelled). Moreover, cells without classification are not admitted by our ML algorithm.

## 3. Results

### 3.1. Training of Morphological Single Cell Analysis

**Figure 3:**
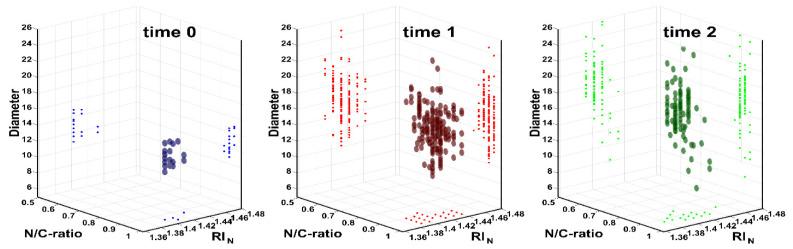
Multiple properties plot of cells from training sample over time (0, 2 and 7 weeks) are reported. Each time step is differently colored for easier readability (blue, 0 weeks with 18 cells, red for training sample at 2 weeks with 191 cells and green for training sample at 7 weeks with 111 cells).

Our measurement approach permits a fine investigation of morphometric cell features at a sub-micrometric resolution. Optical bright field microscopy cannot achieve such a resolution, whereas other microscopic techniques can (confocal microscopy, scanning electron microscope or transmission electron microscope). However, such microscopic techniques require expansive equipment and complex pre-processing operation, which can destroy a biological sample. Light scattering, instead, emerges as a technique for a label-free, low-cost and fine resolution imaging of cells. In Figure 3, biophysical properties of cells from the training sample over time are reported. It appears clear that all the three cell parameters are affected by the culture time.

The cluster formed by the cells, in fact, gradually moves in the shown 3D plots, occupying a different position. Specifically, the points move along the y-axis (diameter) and along the z-axis (RI_N_). However, biophysical properties of cells detected at time 2 were used as the CTC input for the ML algorithm. We considerate remaining cells at time 2 as CTC because all the other terminal cells that would be present in such a cell sample (RBC, lymphocytes, monocytes and granulocytes) naturally die after a maximum two weeks of culturing. Because such cells are, in fact, in their terminal state, not able to replicate and live (since they are not provided with specific supplements). However, it is also necessary to instruct the ML algorithm with the biophysical properties of all the other possible cell classes it can encounter.

Our research group has collected, over the years, detailed biophysical properties of human physiologic circulating cells with the same measurement approach used in the present work. For instance, Dannhauser et al. [23], we characterized the RBC shape and dimension. The biophysical properties of mononuclear cells, instead, were taken from the data of three subsequent works of Dannhauser et al. [20,21,22], in which we characterized human monocytes and lymphocytes, with a clear and precise distinction between lymphocytes subclasses (B-lymphocytes versus T-lymphocytes). Out of this work, we used 110 RBC, 100 T-lymphocytes, 100 B-lymphocytes, 148 monocytes and 136 granulocytes together with the pattern of 111 CTC. The exact values of biophysical properties of such cells are summarized in Table 1.

**Figure 4:**
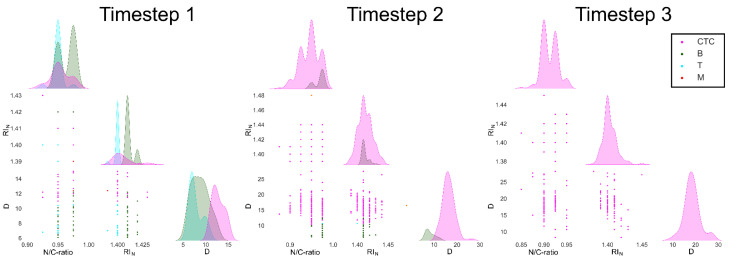
Multi-plot graphs show number and characteristics of each cell type classified by the ML algorithm for NSCLC training sample. Each multi-plot is composed of six different plots: three histogram distribution plots and three scatterplots. Going from upper to lower, left to right: first plot is a histogram plot showing distribution of N/C-ratio values of detected cells; second plot is a scatterplot classifying cell basing on their values of RI_N_ and N/C-ratio; third plot is a scatterplot classifying cell basing on their values of D and N/C-ratio. Fourth plot is a histogram plot showing distribution of RI_N_ values of detected cells; fifth plot is a scatterplot classifying cell basing on their values of D and RI_N_. Sixth and final plot is a histogram plot showing distribution of D values of detected cells.

Such a collection of biophysical properties of different cell classes was used to identify all the mentioned cell types. The trained ML algorithm was then used to implement an automatic recognition of all possible cell types in NSCLC patient samples. We first applied the ML algorithm on the different time steps of the training sample. Results show that the ML algorithm identifies different cell types beyond CTC, as indicated in Figure 4.

Each analysed cell is plotted as function of three biophysical properties. However, in this case, the relationship between the parameters is exploited in six subgraphs. Three of these are distribution graphs, and the remaining three are scatterplot graphs. The distribution graphs show the distributions of the three biophysical properties (RI_N_, N/C-ratio and D), emphasizing the overlapping regions, considering that the values of biophysical properties of different cell types often have similar values. The remaining scatterplot graphs, instead, show all the possible correlations between the three biophysical properties (D vs. N/C-ratio, D vs. RI_N_, N/C-ratio vs. RI_N_).

A colour legend permits us to distinguish the cell classes identified by the ML algorithm. The outcome of time 0 clearly indicates the coexistence of more than one cell type in the NSCLC sample. Four different classes of cells are in fact present: T-lymphocytes, B-lymphocytes, monocytes and CTC. From the histogram plots, we can notice how the N/C-ratio and RI_N_ values of all cells overlap, where CTC can be distinguished because of their significantly higher D values. Values of the N/C-ratio span from 0.92 to 0.98. For time step 1, it is possible to notice that CTC (purple circles) become the predominant cell type. It is also clear how monocytes that were evident at time 0 diminish. A new cell type appears in the obtained results, which can be identified as granulocytes.

It is indeed well known that macrophages are derived from monocytes that, after extravasation, become resident. Furthermore, D values of CTC increase according to the previous time step (D ≤ 20 µm). Values of the N/C-ratio are similar to the ones of time 0. Finally, for time 2, CTC are the only cells that can be found, where all the other cell types diminish. Furthermore, D-values of CTC significantly increase compared to the previous time steps. We also notice a N/C-ratio reduction from time 1 to time 2. On the overall, such a multigraph gives a clear overlook of the three different time steps of the NSCLC training sample. 

In fact, from the beginning to the end of the cell culture time we noticed a gradual overtake of the CTC over the other cell types. Moreover, an interesting change of CTC biophysical properties happens, and in particular, CTC become bigger, with a less representative nucleus. In detail, biophysical properties of CTC have the following (average) values reported in Table 1. In Table 2, the percentage of each identified cell type are summarized for the NSCLC training sample.

### 3.2. ML Results for NSCLC Samples

**Figure 5:**
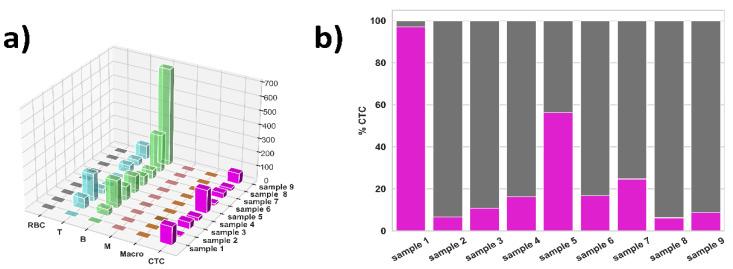
NSCLC sample outcome. (**a**) Dimensional view of each cell classified with the trained ML algorithm (x-axis) in each experiment (y-axis). Z-axis indicates the number of identified cells. Please note that counting of cells refers to an average value of 50 mL of pleural washing, as described in Section 2.2. Each coloured line on x-axis represents a cell type, where y-axis indicates each experiment performed. Height of each bar indicates the number of cell type identified by the ML algorithm. CTC are fully coloured for clarity, where other cell types are transparent. (“RBC” = Red Blood Cells, “T” = T-lymphocytes, “B” = B-lymphocytes, “M” = Monocytes, “Macro” = Granulocytes, “CTC” = Circulating Tumour Cells). (**b**) Bar plot highlights the number of CTC in each NSCLC experiment.

We applied the trained ML algorithm to nine NSCLC samples (sample 1–9). Measurement results are presented in Figure 5 and Table 3, which shows the distributions of the detected cell types.

A minimum amount of RBC, monocytes and macrophages was detected in all NSCLC experiments, whereas the quantity of lymphocytes (both T and B) is variable. Overall, both T- and B-lymphocytes represent the majority of cell classes in all experiments. CTC are also highly represented. However, the variability among all the samples is very high. In detail, we can notice that adenocarcinoma samples (sample 3–9) present a consistent number of lymphocytes. B-lymphocytes represent ~59.87% of all cells, while T-lymphocytes represent ~20.00%. Both monocytes and macrophages are almost absent (respectively ~0.12% and ~0.08%).

The number of CTC in such samples were significant, with an average of ~19.93%. This outcome implies that one cell in five is identified as CTC. For sample 2, which, in addition to NSCLC, the subject also presented a Hodgkin Lymphoma, the number of B-lymphocytes, T-lymphocytes and monocytes is comparable to the one of the adenocarcinoma samples (respectively ~34.87%, ~57.97% and 0.00%), with yet another inversion of lymphocyte subclasses is noticed: in this sample T-lymphocytes are more representative than B-lymphocytes, exactly the opposite in adenocarcinoma samples. Finally, in sample 1, which is a case of NSCLC presenting an infiltrating carcinoma, we detected a great majority of CTC (97.06%), with a very low concentration of B-lymphocytes (2.94%) and no other types of cells.

## 4. Discussion

Lung cancer represents the third most common malignant neoplasia in Europe and represents 85–90% of all malignant lung neoplasia [1,5]. In particular, with the rapid growth of NSCLC cases during these years, new prognostic and diagnostic tools need to be provided in order to reduce cancer mortality. Therefore, discovering associations between CTC from liquid biopsy and cancer is as powerful and significant as a new efficient method to detect them. In most patients, NSCLC is diagnosed after a long medical examination performed from the onset symptoms, such as persistent cough, a non-resolving lung infection, dyspnoea, presence of blood in the sputum, etc. [2]. Moreover, to be diagnosed, NSCLC needs diagnostic imaging and, in the end, a biopsy sample to determine the stage of tumour progression. A reliable method for a fast diagnosis of NSCLC would greatly help in improving therapeutic success [14].

A new idea is here proposed: a morphological single-cell analysis approach combined with a microfluidic device for liquid biopsy. The investigation of a NSCLC sample at different culturing times gave us the possibility to understand the evolution of different cell types and their morphological changes, making CTC predominance against all other cell classes visible. Morphological single-cell analysis followed by machine learning gave us a predictive picture for an NSCLC patient, showing that it is possible to distinguish CTC from other cells. Our measurement approach is fast, reliable, label-free and moreover identifies and counts CTC in a biological fluid. As of today, CTC identification requires long preparing procedures, which often destroy the measurement sample too. The ML algorithm was tested for nine NSCLC patient samples, where the obtained outcome gave evidence that this method has a high specificity and accuracy of detection for each specific type of tumour.

Furthermore, the morphological analysis of cancer cells shows that, once they undergo transformation, they drastically change their phenotype [24]. In fact, the transformation in cells is very useful to be detected, giving the possibility to intercept every trace of cell transformation, even in the early stages of the disease. Such a possibility of a diagnosis of cell transformation in a short time, still favourable for the success of therapeutic treatments, is the main goal of any clinician [24,25].

This represents a pilot study that has highlighted a diagnostic tool for the identification of CTC cells through a precise definition of biophysical parameters. Subsequent studies will be necessary for the evaluation of this technique of CTC directly in peripheral blood. The proof of concept of this study was to identify the presence of CTC in the pleural washing of NSCLC patients for setting up a potential useful diagnostic tool in peripheral blood sampling. This methodology could be used in the future for an assessment of CTC in the bloodstream, that, in this way, compared to pleural washing, could be collected quickly and non-invasively as supplementary laboratory analysis in the diagnosis of suspected lung cancer.

## 5. Conclusions

The results of this work pave the way for positive developments in the diagnostic tumour field. This non-invasive and label-free diagnostic approach, in fact, can provide useful information and help to overcome the challenge of the last decade that sees liquid biopsies as protagonists of a successful but not yet maintained therapeutic promise in the field of lung cancers. The aim of this study is to create a new way of approaching the diagnosis and therapy of lung cancer patients. The here-presented technique offers a deeper and customized picture of each patient, permitting faster targeted diagnosis that should improve patient chances of survival. No less important is considering the reduction in costs that could allow this method, comparing it to current diagnostic techniques (Screen cell, CellSearch, ISET). Due to the sensitivity of the test, it could be also useful in the choice of different therapies based on the detection of different clinical picture. Prospective studies are needed to confirm this finding. To evaluate the clinical relevance, further studies will be necessary to assess relapse-free survival (RFS) after surgical resection to predict survival after relapse (SAR) and overall survival (OS) in NSCLC patients. To conclude, this measurement approach will allow a more effective diagnosis compared with the currently used staging systems with a consequent reduction in the number of therapeutic failures. 

## Figures and Tables

**Table 1:** Values of biophysical properties of physiological PBMC (values from references) and CTC (experiment of this work).

Reference	Cell Type	D [µm]	RI_C_	RI_N_	N/C-Ratio
	*CTC*	*18.44 ± 3.49*	*1.36*	*1.40*	*0.920*
[23]	RBC	7.64 ± 0.91	1.40	-	1.00
[21]	T	6.60 ± 0.36	1.36	1.40	0.950
[20]	B	7.42 ± 0.51	1.36	1.42	0.975
[20]	M	9.57 ± 1.02	1.36	1.38	0.784

**Table 2:** Percentage of each cell types at different time steps of the NSCLC training sample.

	RBC (%)	T (%)	B (%)	M (%)	Macro (%)	CTC (%)
***Time step_1***	*0.00*	*25.71*	*47.14*	*1.43*	*0.00*	*25.71*
***Time step_2***	*0.00*	*0.46*	*11.06*	*0.00*	*0.46*	*88.02*
***Time step_3***	*0.00*	*0.00*	*0.00*	*0.00*	*0.00*	*100.00*
	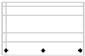	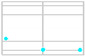	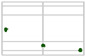	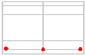	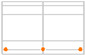	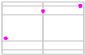

**Table 3:** Cell type found on each of the nine samples.

	Sample 1	Sample 2	Sample 3	Sample 4	Sample 5	Sample 6	Sample 7	Sample 8	Sample 9
**RBC**	**1**	**0**	**0**	**0**	**0**	**0**	**0**	**0**	**0**
**T**	**0**	**80**	**201**	**37**	**4**	**13**	**55**	**44**	**101**
**B**	**3**	**48**	**201**	**103**	**125**	**72**	**64**	**282**	**700**
**M**	**0**	**0**	**1**	**0**	**1**	**0**	**0**	**1**	**0**
**Macro**	**0**	**1**	**0**	**0**	**1**	**0**	**0**	**0**	**2**
**CTC**	**132**	**9**	**49**	**27**	**168**	**17**	**39**	**21**	**77**

## Data Availability

Data are contained within the article.
